# Accessory Ligament of the Deep Digital Flexor Tendon of the Horse Forelimb and Its Relationship with the Superficial Digital Flexor Tendon: A Plastination, Histological, and Morphometry Study

**DOI:** 10.3390/ani14202952

**Published:** 2024-10-14

**Authors:** Gulsum Eren, Octavio López-Albors, Ruth Guilabert Segura, Joana Jordan Montesinos, Rafael Latorre

**Affiliations:** 1 Department of Anatomy and Comparative Pathological Anatomy, Veterinary Faculty, University of Murcia, 30100 Murcia, Spain; ruth.guilabert@um.es (R.G.S.); joanagraya@gmail.com (J.J.M.); latorre@um.es (R.L.); 2 Department of Anatomy, Veterinary Faculty, University of Uludag, Bursa 16059, Türkiye

**Keywords:** horse, superficial digital flexor tendon, deep digital flexor tendon, accessory ligament, E12 sheet plastination, cross-sectional area

## Abstract

**Simple Summary:**

Numerous studies have examined the accessory ligament of the deep digital flexor tendon (AL-DDFT), focusing on both its normal and pathological anatomy. However, limited information is available regarding the precise relationship between the AL-DDFT and its surrounding structures. Clinically, this ligament is often implicated in desmitis and the formation of adhesions, yet the mechanisms driving these pathological changes are not fully understood. While previous investigations have suggested potential contributing factors, detailed descriptive studies are still necessary. In this study, macroscopic, microscopic, and morphometric analyses of the AL-DDFT were carried out. Through dissection and examination of E12 plastinated sections, we provide novel insights into the structural organization and interactions of the AL-DDFT with adjacent tissues. These findings enhance the current understanding of the anatomical and clinical aspects of the AL-DDFT, building upon and integrating previous research.

**Abstract:**

The accessory ligament of the deep digital flexor tendon (AL-DDFT) plays a crucial role in the stay apparatus of the horse. This study aimed to investigate the anatomical relationship between the AL-DDFT, the superficial digital flexor tendon (SDFT), and other structures in the metacarpal region. Sixteen distal forelimbs from eight horses, aged 1 to 6 years, were evaluated through macroscopic, microscopic, and morphometric analyses, utilizing detailed dissection, E12 plastinated sections, and histological analysis. During lateral dissection, a connection was observed between the AL-DDFT and the SDFT. Histological evaluation revealed that this connection was a fibrous band (FB), extending the common synovial sheath (CSS) to the SDFT, along with associated collagen fibrils of the epiligament and peritenon. Additionally, two distinct forms of the AL-DDFT were identified, Type I and Type II, with Type II showing a greater cross-sectional area (CSA) than Type I. While numerous morphological and morphometric studies have explored the AL-DDFT and related structures, research incorporating plastination-based morphological and histological evaluations remains scarce. The findings provide valuable insights for both the morphological and clinical assessment of structures within the metacarpal region.

## 1. Introduction

The flexor tendons and ligaments located in the metacarpal region, the superficial digital flexor tendon (SDFT), the deep digital flexor tendon (DDFT), the accessory ligament of the DDFT (AL-DDFT), and the suspensory ligament (SL), have an essential role in carrying the body weight and in stabilizing the carpus, fetlock, and digit during movement and rest [1,2]. This stabilizing function is further supported by the metacarpal flexor retinaculum (MFR), which, along with the deep fascia, aids in resisting gravitational forces and transmitting muscle force during locomotion [3]. The tendons and ligaments are surrounded and lubricated by the common synovial sheath (Vagina synovialis communis mm. flexorum) (CSS) [2,4,5,6]. The accessory ligament of the deep digital flexor tendon (AL-DDFT), also known as the inferior check ligament (ICL), is a key component of these structures that passively bears the load during extension of the metacarpophalangeal and interphalangeal joints, thereby preventing overstretching of the deep digital flexor tendon (DDFT) [2,4,7].

Although there are studies on the normal or pathological anatomy of AL-DDFT, macroscopic and microscopic information about its relationship with the flexor muscles and CSS is still limited [2,8,9,10,11,12,13,14]. Denoix [2] mentioned that, at the lateral and medial aspects of the DDFT, several fibrous bundles of the AL-DDFT join the SDFT, causing a predisposition to adhesions between them. Magnetic resonance imaging studies revealed a fibrous band extending from the AL-DDFT to the SDFT on the lateral side of the metacarpal region [8]. The presence of such a fibrous structure is biomechanically significant, as it may not only influence the movement of healthy horses but also have clinical implications [2,8,15]. However, there is no mention of it, either in the Nomina Anatomica Veterinaria or in traditional textbooks of horse anatomy. In particular, its proximodistal extension, cross-sectional morphology, tissue composition, and relationship with adjacent structures, such as the MFR and the CSS, deserve further investigation.

Tendon and ligament injuries and their impact on nearby structures are common in sport horses. Ultrasound evaluation is a valuable diagnostic method for identifying these conditions. It is essential to identify infrastructures, and it also helps to assess the cross-sectional area (CSA) of tendons [16,17,18,19,20,21]. To aid in the diagnosis of clinical conditions, establishing CSA values of these structures in healthy animals is also relevant. Additionally, it has been proposed that the evaluation of tendonitis should include an analysis of the ratios between different tendons [17,22,23,24]. For this reason, accurate measurement and calculation of the CSA ratios of the AL-DDFT and its surrounding structures may have an impact in clinical practice.

Sheet plastination of anatomical slices with the epoxy technique (E12) Biodur^®^ (Biodur Products GmbH, Heidelberg, Germany) [25,26] is a unique technique in anatomical research used to prepare thin and transparent sections for evaluating mesoscopic anatomy. This is particularly useful for studies of the connective tissue of the locomotor apparatus, as it has already been published in horses and other species [2,8]. The reasons for this are that (i) all anatomical structures of E12 plastinated slices are kept in their original positions, which gives accurate topographical information, (ii) tissue shrinkage is minimal, as fixatives are not used during this process and epoxy resin is only marginally affected by polymerization, (iii) connective tissue becomes transparent, so fibrous structures such as fascia, tendons, periosteum, synovial sheaths, etc. are well delimited from the surrounding tissues, and (iv) after epoxy curing, the connective tissue is autofluorescent, and for this reason it can be directly viewed under fluorescent or confocal microscope without any staining [25,26].

Given the clinical significance of the AL-DDFT, this study aimed to enhance the understanding of its detailed anatomy from both a topographical and morphometric perspective. Particular attention was paid to the aforementioned fibrous band connecting the AL-DDFT to the SDFT in terms of shape, size, proximodistal extension, and relationship with the MFR and CSS. Furthermore, direct measurements from E12 plastinated slices of the AL-DDFT and neighboring structures along the proximal metacarpal region brought accurate outcomes that might be of clinical relevance in equine practice.

## 2. Materials and Methods

Forelimbs from eight horses *(n* = 16) cut at the level of the antebrachiocarpal joint were obtained from a local abattoir. Neither sex nor exact age were available, but from approximate estimation of the teeth, they were 1 to 6 years old. All horses had no diagnosed problems in the locomotor system, and none of them were dedicated to any particular athletic performance, as they were raised as livestock animals in farms in the region. The samples (distal limbs) were transported to the dissection room of the Veterinary Faculty (University of Murcia) within a maximum time of 2 h after slaughter. Upon arrival, the limbs were thoroughly washed and the joints placed in anatomical position, particularly the metacarpalphalangeal joint, which was positioned by attaching a wire from the dorsal aspect of the metacarpal bone III to the middle dorsal point of the borde solearis of the hoof. Ten limbs were immediately frozen at −20 °C for 7 days, while the remaining limbs (*n* = 6) were immersed for 4 weeks in 10% formaldehyde solution.

### 2.1. Dissection

Dissection of the metacarpal region was performed on six limbs from a medial to lateral, palmar to dorsal, and superficial to deep approach. In addition to the SDFT, DDFT, AL-DDFT, and SL, careful dissection was performed to visualize and understand the close relationship among them, the CSS, nerves, and vessels. Particular attention was paid to the lateral side to understand the topography of the AL-DDFT and its potential connections with the SDFT, DDFT, and CSS.

### 2.2. Cryosectionning

**Figure 1:**
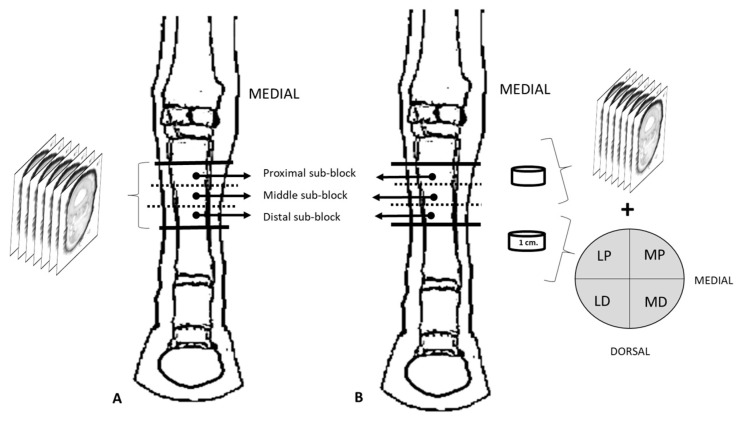
Dorsal view of the distal limbs from which the sections were obtained for plastination (**A**) and plastination and histology (**B**). LD. Laterodorsal; LP. Lateropalmar; MD. Mediodorsal; MP. Mediopalmar.

One week after initial freezing, the limbs (*n* = 10) were released from the wires used to maintain correct joint positioning and transferred to a deep freezer (−76 °C), where they were stored for a minimum of 10 days. A block was obtained from each limb with a high-speed band saw from 3 cm distal from the proximal border of the metacarpal bone III to 10 cm proximal to the proximal sesamoid bones. Each block was then divided into approximately equal sub-blocks named proximal (P), middle (M), and distal (D) for further processing. Six limbs were used for epoxy E12 plastination (Figure 1A), and the remaining four limbs for both epoxy plastination and histological study (Figure 1B).

#### 2.2.1. Plastination

P, M, and D sub-blocks for plastination (*n* = 30, 3 sub-blocks × 10 limbs) were serially cross-sectioned with a band saw (Figure 1A). All sections were of 1–2 mm thickness, except those aimed at histological staining, which were 1 cm thick (see Section 2.2.2, Figure 1B). The sections were immediately immersed in cooled acetone (−10 °C), and the surfaces of the slices were gently cleaned with a brush. A total of 156 sections was obtained (15–26 sections per limb). Packages including 8 or 9 sections from the labeled P, M, and D sub-blocks of the same limb were mounted separately and transferred to a bath of cooled pure acetone (−15 °C) for further plastination processing (impregnation and curing), as it is described by Sora et al. [25].

#### 2.2.2. Histological Processing

A cross-section of 1 cm thickness was obtained from the middle point in P, M, and D sub-blocks [*n* = 12, 3 sub-blocks × 4 limbs]. Sections were left to thaw, and then the tissues in palmar position to the metacarpal bone III (SL, AL-DDFT, DDFT, SDFT, and CSS) were isolated and divided into four equal quadrants, named laterodorsal (LD), lateropalmar (LP), mediodorsal (MD), and mediopalmar (MP) samples (Figure 1B). After fixation for 2 weeks in 10% paraformaldehyde at 4 °C, the samples [*n* = 16] were routinely processed for paraffin embedding, microtome sectioned (10 microns thickness), and finally stained with Gomori trichrome.

#### 2.2.3. Microscopy Viewing

Plastinated sections were viewed with a light stereo microscope (Leica MC170 HD^®^, Leica Microsystems GmbH, Germany) equipped with a digital camera. As is currently known, E12 plastinated slices exhibit autofluorescence. Utilizing this property, selected epoxy plastinated sections were evaluated using a Leica Thunder-TIRF^®^ imager Widefield Microscope (Leica Microsystems, Wetzlar, Germany). Histological slides were viewed with a light microscope (Zeiss Axioskop 40^®^, Carl Zeiss, Jena, Germany) equipped with a digital camera.

### 2.3. Morphometry

Epoxy plastinated sections were also used for morphometry. Sections from P, M, and D sub-blocks of each limb were scanned with an Epson Expression 1680 Pro scanner at a resolution of 3200 dpi for morphometric analysis. The cross-sectional areas of the SL, AL-DDFT, DDFT, SDFT, and metacarpal bones were measured using Sigma Scan Pro5 software (Image Analysis Version^®^ 5.0.0, Systat Software Inc., San Jose, CA, USA). All measurements were performed by the same researcher. Metric data were recorded as an Excel file (Microsoft Office^®^ 2011, Microsoft, Redmond, WA, USA) for statistical analysis.

### 2.4. Statistical Analysis

Morphometry data were processed with the SPSS 24.0^®^ (IBM, SPSS Inc., Chicago, IL, USA) statistical package. Descriptive statistics, including arithmetic mean and standard deviation, were obtained. ANOVA was used to evaluate the associations between the cross-sectional areas of the AL-DDFT, DDFT, SDFT, SL, metacarpal bone III, metacarpal bone II, and metacarpal bone IV and the independent variables (factors). These factors were the limb sub-blocks (proximal, middle, and distal), the cross-sectional shape of the AL-DDFT (Type I and Type II, as described in the results), and the forelimb side (right or left). Prior to ANOVA, the Kolmogorov–Smirnov test for normality and the Levene test for homogeneity of variances were performed (*p* > 0.05). The significance level for ANOVA was set at 95% (*p* < 0.05).

## 3. Results

### 3.1. Dissection

**Figure 2:**
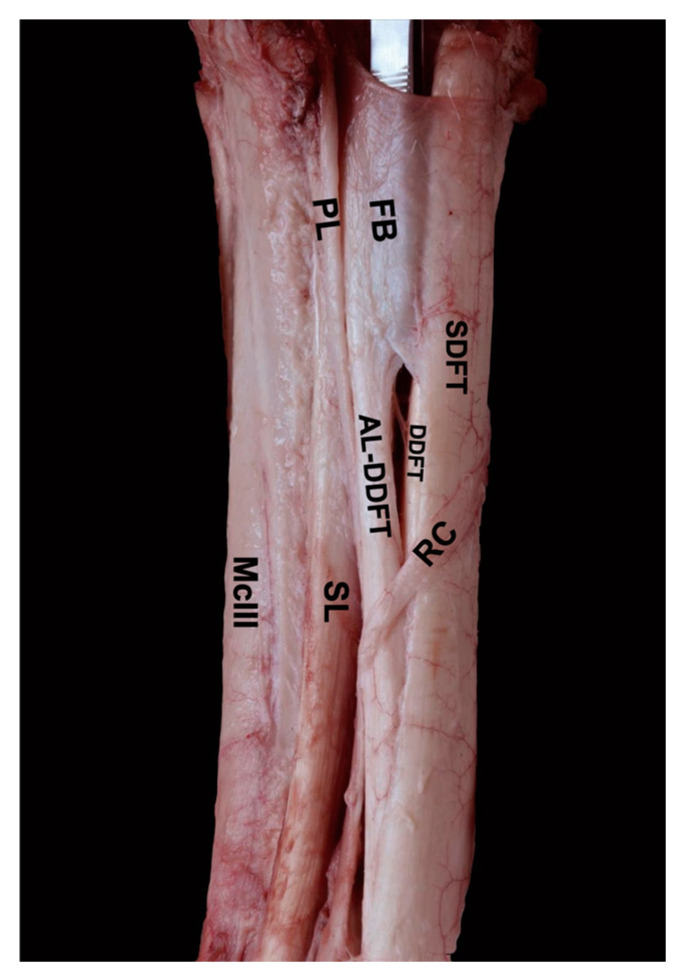
Detailed dissection of the proximal metacarpal region, lateral view. The structures are located in their appropriate anatomical positions. The fibrous band (FB) extending from the AL-DDFT to the SDFT is clearly identified. SDFT. Superficial digital flexor tendon; DDFT. Deep digital flexor tendon; AL-DDFT. Accessory ligament of the DDFT; SL. Suspensory ligament; PL. Lateral palmar nerve; RC. Communicating branch; McIII. Third metacarpal bone; FB. Fibrous band between AL-DDFT and SDFT.

Following dissection of the skin of the palmar aspect of the metacarpal region, the MFR appeared as a dense connective band attached to the metacarpal bones II and IV, encircling the flexor tendons. The dissection through the MFR and the CSS allowed access to the DDFT, SDFT, SL, and AL-DDFT (Figure 2). The lateral dissection of the proximal third of the metacarpal region revealed that the MFR has a thickened band of connective tissue extending from the AL-DDFT to the SDFT. This fibrous band (FB) gradually increased in thickness toward its distal end, at approximately 3–4 cm proximal to the ramus communicans of the palmar nerves, and became clearly visible when the SDFT was displaced palmarly. In contrast, dissection of the metacarpal flexor retinaculum (MFR) on the medial side did not reveal any significant thickening along its proximodistal extent. Regarding the CSS, it extended among the SDFT, DDFT, and AL-DDFT, with a synovial compartment between the DDFT and SDFT ending at approximately 3 cm distal from the antebrachiocarpal joint.

### 3.2. Cross-Sectional Anatomy

**Figure 3:**
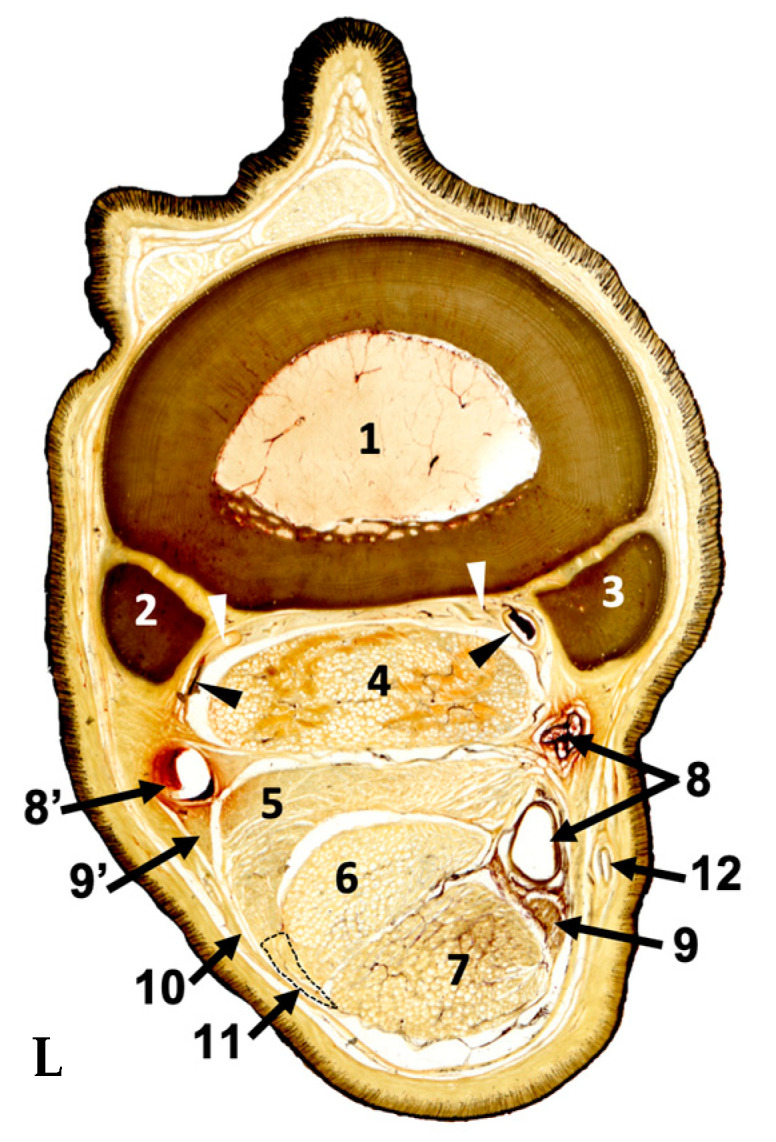
Plastinated cross-section from a distal (D) sub-block with Type II AL-DDFT. Since tissue shrinkage is minimal, anatomical structures are located in their original positions. L: Lateral aspect; 1. Metacarpal bone III; 2. Metacarpal bone IV; 3. Metacarpal bone II; 4. Suspensory ligament; 5. AL-DDFT: Accessory ligament of the DDFT; 6. DDFT: Deep digital flexor tendon; 7. SDFT: Superficial digital flexor tendon; 8. Arteria and vena digitalis palmaris communis II; 8’. Arteria digitalis palmaris communis III; 9. Medial palmar nerve; 9’. Lateral palmar nerve; 10. Metacarpal flexor retinaculum (MFR); 11. Fibrous band (outlined); 12. Cephalic vein; Black arrowheads: palmar metacarpal artery and vein; White arrowheads: medial and lateral palmar metacarpal nerve.

Plastinated slices were serially evaluated from proximal to distal sub-blocks. The following anatomical structures were identified in full detail: the metacarpal II, III, and IV bones; the MFR, DDFT, SDFT, SL, AL-DDFT, and CSS; the arteries and veins digitalis palmaris communis II and digitalis palmaris communis III; palmar metacarpal arteries and veins; and lateral and medial palmar nerves (Figure 3).

**Figure 4:**
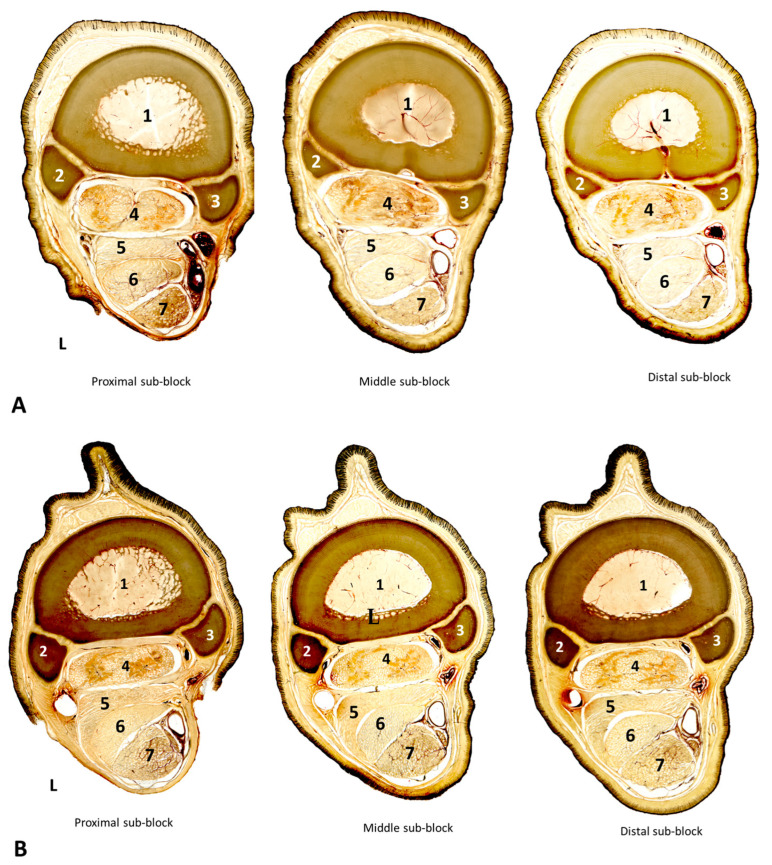
E12 plastinated sections from proximal (P), middle (M), and distal (D) sub-blocks of the metacarpal region. Scanned plastinated sections comparing the two morphological types of the accessory ligament of the deep digital flexor tendon (AL-DDFT) and their relationship with adjacent structures. (**A**) Type I AL-DDFT, (**B**) Type II AL-DDFT. L: Lateral aspect, 1. Metacarpal bone III, 2. Metacarpal bone II, 3. Metacarpal bone IV, 4. Suspensory ligament (SL), 5. Accessory ligament of the DDFT (AL-DDFT), 6. Deep digital flexor tendon (DDFT), 7. Superficial digital flexor tendon (SDFT). Type I AL-DDFT has an ellipsoid cross-sectional shape in the proximal block, gradually turning into a “crescent” shape in the distal blocks. Type II AL-DDFT has a distinct “crescent” shape in all blocks from proximal to distal. In Type I, the rectangular shape of the SDFT changes as it progresses from P to the lower blocks, developing a distinct horn-like dorsolateral projection. In contrast, in Type II, the SDFT showed a more rounded shape along its proximodistal levels, and dorsolateral horn-like projection was not observed.

The morphology and structure of the AL-DDFT were depicted in detail in the plastinated sections. Two morphologically different types of AL-DDFT, named Type I and Type II, were identified in the whole series of plastinated cross-sections (Figure 4). Type I had an ellipsoid cross-sectional shape in the proximal third of the AL-DDFT (P sub-blocks), which progressively changed in the M and D sub-blocks into a rather “crescent” shape due to a horn-like lateral projection interposed between the dorsolateral corner of the DDFT and the MFR. Type II morphology showed a marked “crescent” shape all along the P, M, and D sub-blocks of the AL-DDFT. In this type, the aforementioned horn-like lateral projection was more evident, such that it not only separated the DDFT from the MFR but even palmarly reached the dorsolateral corner of the SDFT. In both types, the apex of the horn-like lateral projection of the AL-DDFT and SDFT tended to fuse with the fibrous fascicles of the MFR (laterally), and to some extent the CSS (medially), hence creating a fibrous band (FB), which was remarkably thinner and longer in Type I than in Type II samples. Type I was found in six out of the 10 forelimbs, while Type II conformation was present in the remaining four forelimbs, without any difference between the right and left limbs of the same horse.

In accordance with the different cross-sectional shapes of the AL-DDFT (Types I and II), the morphology of the SDFT was also different. In Type I, the SDFT cross-section was nearly rectangular in P sub-blocks but progressively developed a prominent horn-like dorsolateral projection in M and D sub-blocks (Figure 4A). In contrast, in Type II, the SDFT was more rounded all along its proximodistal distance, since no clear dorsolateral horn-like projection was observed (Figure 4B). The changes in the cross-sectional morphology of the AL-DDFT and the SDFT in Types I and II also influenced the DDFT shape, which was a bit less flattened in Type I than in Type II samples.

**Figure 5:**
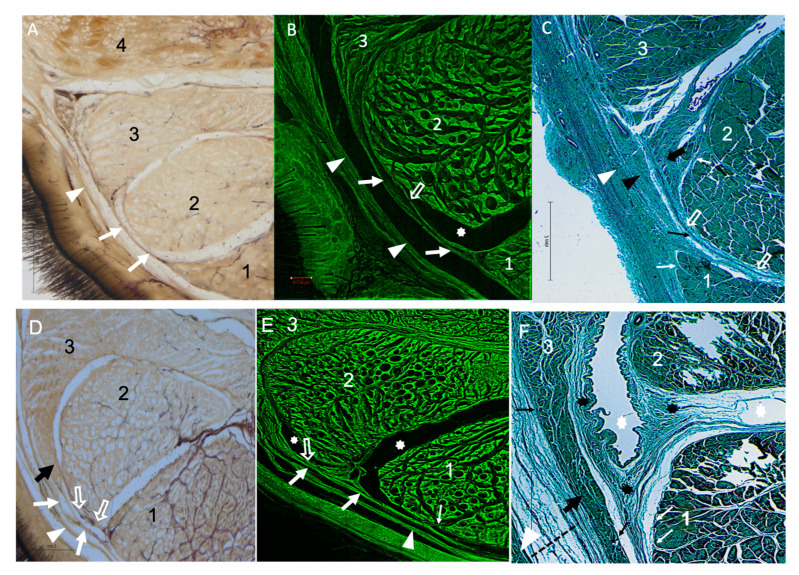
Cross-sections from the metacarpal region, focused on the lateral relationship between the AL-DDFT and SDFT. Type I AL-DDFT (**A**–**C**) and Type II AL-DDFT (**D**–**F**). (**A**,**D**) Bright field view of E12 plastinated sections. (**B**,**E**) Fluorescent view of E12 plastinated sections. (**C**,**F**) Bright field microscopy view of histological sections. White arrows: Fibrous extension between the AL-DDFT and SDFT; Black arrows: Horn-like projection of the AL-DDFT; White arrowheads: Metacarpal flexor retinaculum (MFR); Black arrowhead (**C**) and dashed line (**F**): Deep fascia; Open arrow: Synovial sheath; White asterisks: Synovial space; Black asterisks: Synovial membrane lining the medial surface of the AL-DDFT. Thin black arrow: Epiligament; Thin white arrow: Peritenon; 1. Superficial digital flexor tendon (SDFT); 2. Deep digital flexor tendon (DDFT); 3. Accessory ligament of the DDFT (AL-DDFT); 4. Suspensory ligament (SL). The fibrous band (FB) extending from Type I is seen as a more distinct extension. The Type 2 AL-DDFT has a “crescent” shape, and the lateral edge extends more toward the SDFT in the palmar direction, so the FB appears shorter than Type I. (**A**,**D**). It can be observed that the medial surface of the AL-DDFT and FB are lined with the synovial sheath and the synovial layer (**C**,**F**). Where the FB connects to the SDFT, a synovial gap is seen between the two structures, which in Type 2 is seen as a synovial space between the FB and the DDFT extending from the AL-DDFT (**B**,**C**,**E**,**F**).

The microscopic analysis of the plastinated and histological samples revealed further detailed information on the FB (Figure 5). Structurally, it consisted of several fascicles of dense connective tissue arising from and connecting the respective horn-like projections of the AL-DDFT and SDFT. The horn-like projections of AL-DDFT and SDFT were well differentiated from the connective fascicles, as the former showed typical histological features of tendinous tissue with intermingled myofibroblasts, while the connective fascicles were the epiligament. Such bundles of connective tissue (epiligament and peritenon) displayed a core of well-organized collagen fibers, with outer and inner layers of loose connective tissue bands, which included elastic fibers and vessels. Under fluorescent confocal view (Figure 5B,E), the bundles of connective tissue of the epiligament and peritenon coming from the respective horn-like projections of the AL-DDFT and SDFT were seen to fuse laterally to the MFR and medially to either the CSS or, occasionally, the DDFT. Careful examination of the plastinated and histological samples revealed variable contribution of the AL-DDFT to the FB between Type I and Type II samples. In Type I samples, the contribution of the horn-like projection of the AL-DDFT to the FB was relatively discrete, resulting in a long and thin FB of connective fascicles between the AL-DDFT and SDFT. In contrast, in Type II samples, the horn-like projection of the AL-DDFT was longer, with the FB forming from the fusion of the connective fascicles of the AL-DDFT with the MFR and extending from here to the lateral aspect of the SDFT. Hence, in Type II samples, the FB was remarkably thicker. All these features were more evident in M than in P and D sub-blocks, hence demonstrating that the thickest development of the FB was located at approximately 7–10 cm distal from the antebrachiocarpal joint. On the other hand, no differences between the left and right limbs of the same animal were observed.

### 3.3. Morphometric Analysis

In limbs on both sides, all measured structures were found to be similar at the three sub-blocks levels (P, M, D) (*p* > 0.05) (Table 1). The largest structure at all levels was the SL, followed in decreasing order by the DDFT, the AL-DDFT, and the SDFT. The AL-DDFT cross-sectional area was found to differ according to its morphology, with Type II being remarkably bigger than Type I at all levels (P, M, D) (*p* < 0.001). In addition, the whole AL-DDFT area decreased from the P to the M levels in Type I but did not vary in the proximodistal direction in Type II (Table 1). The cross-sectional areas of the DDFT, SL, and AL-DDFT (average Types I and II) were significantly higher in the P and D sub-blocks compared to the M sub-block.

The cross-sectional area ratios between the measured structures are displayed in Table 2. Significant differences were observed between the AL-DDFT/DDFT ratios in Type I and Type II samples, as well as between the AL-DDFT/SL and metacarpal III/AL-DDFT values across all three levels. However, the AL-DDFT/SDFT ratio differed only at the M level.

## 4. Discussion

Previous studies have extensively described the topography of the main structures in the metacarpal region, and our results agree with them [1,2,6,8]. Although the injury history of the horses was unknown, our dissection revealed that the anatomical locations and appearance of the structures were normal, and no abnormalities were detected. Particular attention was paid to a thickened area of the MFR on the lateral side, named the fibrous band (FB). Macroscopic and microscopic examinations of plastinated sections and histological images showed that the FB extended between the AL-DDFT and SDFT, consistent with findings reported in previous studies [2,8]. According to Scalec et al. [3], in the region where the FB is located, the deep fascia is thinned to such an extent that it is difficult to separate it from the MFR, and our results are consistent with this finding. Denoix [2] stated that around this region, various fibrous bundles of the AL-DDFT join the SDFT, which may predispose the area to local adhesions. Our study, in which it is clearly seen that the epiligament of the AL-DDFT extends to the SDFT together with the synovial sheath, is also consistent with this information. Nagy and Dyson [8] reported that in dissections of 10 forelimbs, nine of them showed fibrous bundle connections between the AL-DDFT and SDFT in the lateral aspect. This was also found in 27 out of 29 magnetic resonance images of the left forelimb. Moreover, they also reported that in two dissections and 10 magnetic resonance images, there were fibrous bundles between the AL-DDFT and DDFT. In general terms, our findings are also consistent with these authors [8]. In all the dissections and plastinated sections we studied, there were fibrous bundles between the AL-DDFT and SDFT, and occasionally also between the AL-DDFT and DDFT. However, in addition to Nagy and Dyson [8], we found that the fibrous bundles between the AL-DDFT and DDFT were only found in horses with Type II in both left and right forelimbs. In Type I, the fibrous bundles extended laterally and only between the AL-DDFT and SDFT. Therefore, in magnetic resonance examinations, it might be important to consider the type and shape of the AL-DDFT in cases of fibrous adhesions between the AL-DDFT and DDFT that could be encountered.

The AL-DDFT has been described previously as rectangular in shape in the proximal portion, flattening as it extends distally and ultimately adopting a “C” shape, particularly at the junction with the DDFT. With this form, it has a shape that almost surrounds the DDFT [2,8,15]. The AL-DDFT, which we have defined as Type I in six out of 10 samples, complies with this description. In this study, the AL-DDFT is also described as Type II, with the shape of a “crescent” all along the proximodistal levels, which has not been mentioned before. This shape difference in the AL-DDFT could be of importance, especially in examinations using imaging diagnostic methods and morphological and morphometric measurements, as well as in biomechanical evaluations [7,8,12,13,14,15]. It is reported that when the AL-DDFT is loaded, a compressive force will be created on the DDFT due to wrapping by the AL-DDFT [4,15,27]. Therefore, it could be hypothesized that the Type II AL-DDFT, whose FB tends to laterally surround the DDFT, also has a different biomechanical effect from that of Type I. Additionally, it was observed that the cross-sectional shapes of the SDFT and DDFT appeared to adapt to the differing morphologies of Type I and Type II AL-DDFTs. It is important to estimate the magnitude and distribution of local stresses in these structures by determining the geometries of tendons and ligaments with imaging techniques [28]. These different forms of the AL-DDFT and DDFT can be effective in the loads applied to the ligament and tendon during movement and, hence, in calculations such as Young’s modulus, elastic modulus, or stress performed using imaging techniques [15,28,29,30]. On the other hand, Type II was found to be less dominant, but the limited number of samples used in the study makes it difficult to make definitive comments on this issue. Studies about the relationship between the AL-DDFT and the flexor tendons can be conducted with more material and animals with a well-defined age interval and training history.

Injuries in the SDFT are mostly seen in the mid-metacarpal region. This region has weak synovial and blood supplies [31,32]. Leach et al. [6] reported that there is a strong CSS extension from the DDFT to the SDFT in the carpal canal and that there is a possibility of blood flow in the region through this extension. Similarly, in our study, the vessels in the CSS’s synovial layer covering the medial surface of the FB are clearly visible. This leads us to conclude that the CSS provides a vascular passage between the two structures, which might provide a vascular supply to the SDFT to reduce local stresses or support tendon healing [32]. However, CSS vascularity may also be involved in situations that trigger adhesions in the region. It is reported that the desmitis of the AL-DDFT, one of the most frequently seen problems in horses, generally results in adhesion to surrounding structures. Among these structures, adhesion between the SDFT and AL-DDFT is quite common [6,33,34,35]. One of the reasons for adhesion, which occurs as an undesirable response, especially in tendon healing, is the early inflammatory response that appears to contribute to the healing process due to poor blood supply and low cellularity in the tendons [36,37]. When histological sections were examined in our study, blood vessels in the CSS’s synovial membrane covering the FB’s medial surface were clearly seen. This may accelerate adhesion formation in the case of injury. Additionally, since the AL-DDFT extends more to the SDFT in Type II than in Type I, its connection to the SDFT is seen more clearly than in Type I. This should be taken into consideration when approaching ultrasonography evaluations with suspicion of adhesions. The areas where adhesion is predicted may have an enlargement or tearing in the FB mentioned between the SDFT and AL-DDFT, but it could also be a normal anatomical structure. Therefore, in some cases, it may be misinterpreted as typical adhesions [34,35].

Morphometric measurements were directly obtained from E12 plastinated cross-sections, which minimize positional changes of the structures and shrinkage. [26]. In biomechanical studies, accurate evaluation of the cross-sectional area (CSA) of tendons and ligaments using ultrasounds is essential for identifying potential lesions through comparison with normative data derived from healthy animals [7,20,27,28,38,39,40]. In this study, the highest CSA corresponded to the SL, followed by the DDFT, AL-DDFT, and SDFT, which is consistent with previous studies [41,42]. Also, while the CSA of the DDFT tended to decrease distally, increasing CSA values were found in the SDFT, a result also found in Kathiawadi and Marwadi horses [20,43]. Given that the length and thickness of tendons exhibit variability based on factors such as breed, age, training history, and load on the tendon [13,42,43,44,45,46,47,48,49], it may be more relevant to assess the obtained values by establishing proportions between the measurements to minimize discrepancies among animals and structures. For instance, Yin et al. [15] measured the CSA of the AL-DDFT and DDFT in slaughterhouse forelimb samples, and the CSA values were higher than those from a similar region in our study. However, when the proportions of the CSA values of the AL-DDFT/DDFT in the two studies are compared, pretty similar values are found, which enhances the importance of using ratios rather than absolute values when comparing morphometric studies of the tendons and ligaments.

## 5. Limitations

A limitation of the study is that the limbs used were not standardized in age, size (relatively young animals only), and sex, and their numbers were limited. The study determined that Type II was less frequent, but the limited number of samples also limited the use of a definitive statement on this subject. Another important limitation is that the training history of the horses and their nutrition were also unknown.

## 6. Conclusions

In the anatomical evaluation of the palmar metacarpal region, either by anatomical or imaging techniques, the presence of a FB extending laterally from the AL-DDFT to the SDFT should considered. The thickness of this connection may vary depending on whether the AL-DDFT conformation is Type I or Type II, and this should be considered, especially for diagnostic imaging evaluations. In Type II AL-DDFTs, the FB is more conspicuous than in Type I, which may be important from a biomechanical and clinical perspective. The morphometric information obtained here is relevant for size studies of the DDFT, SDFT, and AL-DDFT since it varies depending on the morphological type of the AL-DDFT and the level of measurement in the distal direction. In future studies, increasing the sample size, particularly in comparative analyses that consider specific age and size categories or healthy versus injured horses, will be beneficial for obtaining more detailed information.

## Figures and Tables

**Table 1:** Cross-sectional area (mean ± standard deviation, mm^2^) of the flexor tendons, ligaments, and metacarpal bones from three sub-blocks.

Structures	Proximal Sub-Blocks	Middle Sub-Blocks	Distal Sub-Blocks
AL-DDFT Type I	73.1 ± 16 ^Aa^	65.2 ± 12.7 ^Ab^	68.7 ± 13.9 ^Ab^
AL-DDFT Type II	110.7 ± 4.8 ^Ba^	105.4 ± 8.7 ^Ba^	106.1 ± 3.4 ^Ba^
AL-DDFT (General)	82.2 ± 21.5 ^a^	75.7 ± 21.3 ^a^	83.7 ± 8.2 ^a^
DDFT	95.5 ± 14.6 ^a^	82.5 ± 11.5 ^b^	80.2 ± 21.6 ^a^
SDFT	71.7 ± 19.9 ^a^	69.1 ± 23.7 ^a^	78.4 ± 21.7 ^a^
SL	157.3 ±21.7 ^a^	148.5 ± 19.3 ^b^	153.2 ± 20.5 ^ab^
Metacarpal bone III	761.8 ± 99.1 ^a^	676.6 ± 79 ^b^	706.4 ± 49 ^b^
Metacarpal bone II	64.6 ± 16.4 ^a^	51.7 ± 11.6 ^b^	46.8 ± 9.9 ^c^
Metacarpal bone IV	66.9 ± 23.5 ^a^	46 ± 15.4 ^b^	38.3 ± 14.4 ^c^

^a,b^ Values with different superscripts in the same row are significantly different (*p* < 0.05, except ^c^ where *p* = 0.05). ^A,B^ Values for Types I and II of the AL-DDFT with different superscripts in the same column are significantly different (*p* < 0.05). SDFT: Superficial digital flexor tendon; DDFT: Deep digital flexor tendon; AL-DDFT: Accessory ligament of the DDFT; SL: Suspensory ligament.

**Table 2:** Size ratios between the flexor tendons, ligaments, and metacarpal bones from three levels.

Structures	Proximal Sub-Block	Median Sub-Block	Distal Sub-Block
AL-DDFT/DDFT			
Type I AL	0.8 ± 0.2 ^Aa^	0.8 ± 0.1 ^Aa^	0.9 ± 0.2 ^Ab^
Type II AL	1.0 ± 0.1 ^Ba^	1.1 ± 0.5 ^Ba^	1.3 ± 0.0 ^Bb^
AL-DDFT/SDFT			
Type I AL	1.2 ± 0.2 ^Aa^	1.2 ± 0.1 ^Aa^	1.1 ± 0.2 ^Ab^
Type II AL	1.1 ± 0.5 ^Aa^	1.0 ± 0.9 ^Ba^	1.0 ± 0.3 ^Ab^
AL-DDFT/SL			
Type I AL	0.5 ± 0.5 ^Aa^	0.4 ± 0.4 ^Aa^	0.4 ± 0.4 ^Ab^
Type II AL	0.7 ± 0.0 ^Ba^	0.7 ± 0.5 ^Ba^	0.7 ± 0.0 ^Bb^
DDFT/SDFT	1.4 ± 0.3 ^a^	1.3 ± 0.3 ^a^	1.1 ± 0.3 ^b^
SL/SDFT	2.3 ± 0.5 ^a^	2.3 ± 0.6 ^a^	2.1 ± 0.6 ^b^
SL/DDFT	1.7 ± 0.3 ^a^	1.8 ± 0.2 ^b^	1.9 ± 0.3 ^c^
Metacarpal bone III/AL-DDFT			
Type I AL	10.5 ± 1.5 ^Aa^	10.2 ± 1.1 ^Aa^	10.4 ± 1.7 ^Aa^
Type II AL	7.3 ± 0.4 ^Ba^	7.1 ± 0.4 ^Ba^	8.8 ± 0.1 ^Ba^
Metacarpal bone III/DDFT	8 ± 0.8 ^a^	8.2 ± 0.6 ^a^	8.9 ± 0.6 ^b^
Metacarpal bone III/SDFT	11.2 ± 2.2 ^a^	10.5 ± 2.3 ^a^	9.6 ± 2.3 ^a^
Metacarpal bone III/SL	4.9 ± 0.5 ^a^	4.6 ± 0.4 ^b^	4.7 ± 0.5 ^b^

^a,b,c^ Values with different superscripts in the same row are significantly different (*p* < 0.05). ^A,B^ Values with different superscripts in the same column are significantly different (*p* < 0.05). SDFT: Superficial digital flexor tendon; DDFT: Deep digital flexor tendon; AL-DDFT: Accessory ligament of the DDFT; SL: Suspensory ligament.

## Data Availability

Data are contained within the article.
